# Basic science methods for the characterization of variants of uncertain significance in hypertrophic cardiomyopathy

**DOI:** 10.3389/fcvm.2023.1238515

**Published:** 2023-08-01

**Authors:** Chang Yoon Doh, Thomas Kampourakis, Kenneth S. Campbell, Julian E. Stelzer

**Affiliations:** ^1^ School of Medicine, Case Western Reserve University, Cleveland, OH, United States; ^2^ Randall Centre for Cell and Molecular Biophysics, and British Heart Foundation Centre of Research Excellence, King’s College London, London, United Kingdom; ^3^ Division of Cardiovascular Medicine, University of Kentucky, Lexington, KY, United States; ^4^ Department of Physiology and Biophysics, School of Medicine, Case Western Reserve University, Cleveland, OH, United States

**Keywords:** hypertrophic cardiomyopathy (HCM), dilated cardiomyopathy (DCM), variants of uncertain clinical significance (VUS), restrictive cardiomyopathy (RCM), high throughput screen (HTS), mathematical modeling & simulation, arrhythmogenic cardiomyopathy, cardiac myosin binding protein C (cMyBP-C)

## Abstract

With the advent of next-generation whole genome sequencing, many variants of uncertain significance (VUS) have been identified in individuals suffering from inheritable hypertrophic cardiomyopathy (HCM). Unfortunately, this classification of a genetic variant results in ambiguity in interpretation, risk stratification, and clinical practice. Here, we aim to review some basic science methods to gain a more accurate characterization of VUS in HCM. Currently, many genomic data-based computational methods have been developed and validated against each other to provide a robust set of resources for researchers. With the continual improvement in computing speed and accuracy, in silico molecular dynamic simulations can also be applied in mutational studies and provide valuable mechanistic insights. In addition, high throughput *in vitro* screening can provide more biologically meaningful insights into the structural and functional effects of VUS. Lastly, multi-level mathematical modeling can predict how the mutations could cause clinically significant organ-level dysfunction. We discuss emerging technologies that will aid in better VUS characterization and offer a possible basic science workflow for exploring the pathogenicity of VUS in HCM. Although the focus of this mini review was on HCM, these basic science methods can be applied to research in dilated cardiomyopathy (DCM), restrictive cardiomyopathy (RCM), arrhythmogenic cardiomyopathy (ACM), or other genetic cardiomyopathies.

## Introduction

Hypertrophic cardiomyopathy (HCM) is a common heart condition with a prevalence of 1:200–500 (1, 2). It is characterized by an increase in left ventricular wall thickness in the absence of abnormal loading conditions and without an identifiable secondary cause such as hypertension or aortic stenosis (3). It is thought to be a result of heterogeneous sets of mutations in sarcomere proteins (4, 5). Since HCM is highly variable in both expressivity and penetrance with many modifying factors (6, 7), the precise genetic determination is important for diagnosis, treatment, and prognosis.

**Figure 1:**
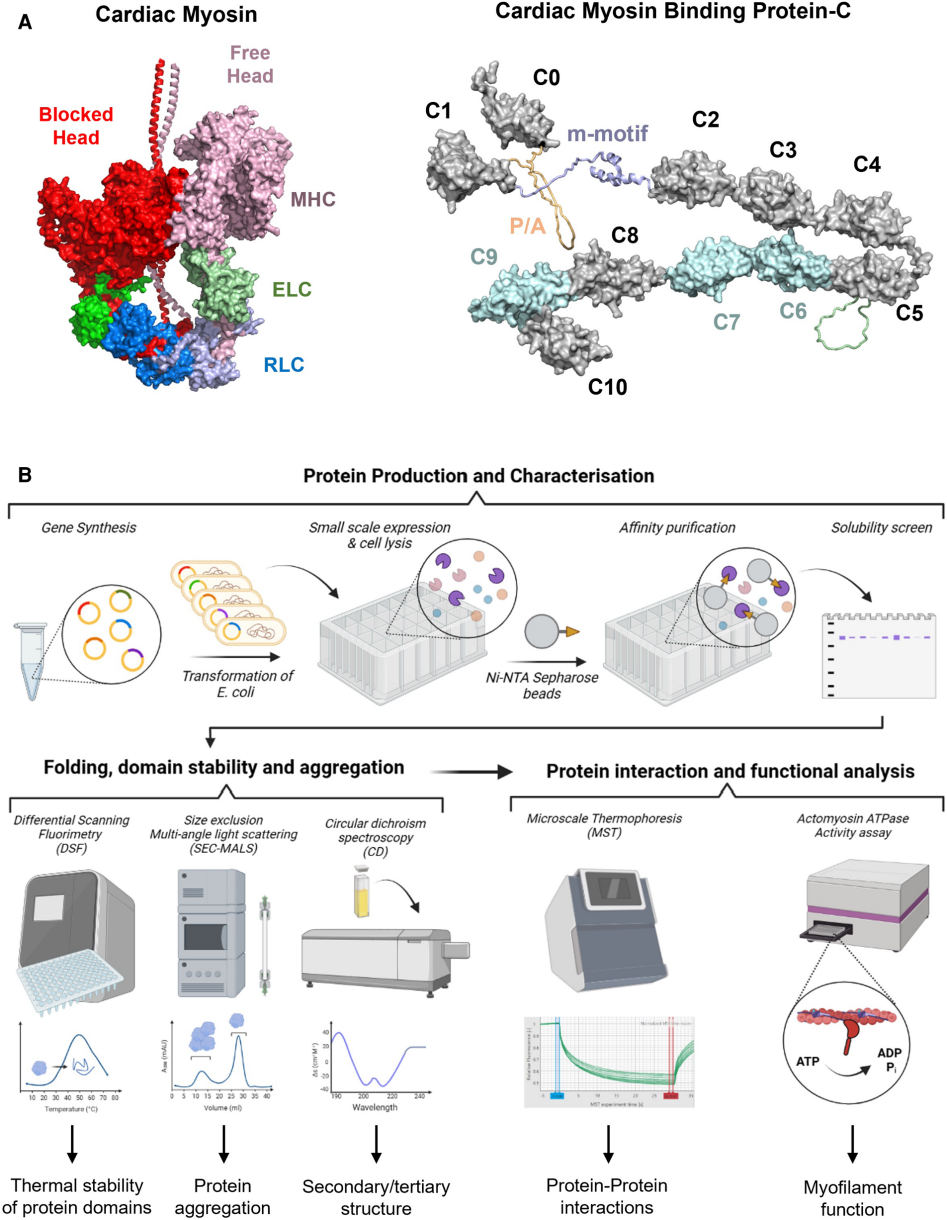
(**A**) Structural models of cardiac myosin folded into the interacting heads motif (left) and cardiac myosin binding protein-C (right). Individual domains are labelled accordingly. RLC, regulatory light chain; ELC, essential light chain; MHC, myosin heavy chain; P/A, proline/alanine-rich linker. (**B**) High throughput *in vitro* screening process for VUS in genes associated with HCM. Gene synthesis and expression allows an initial screen for protein folding and production of purified proteins for characterization. A variety of biophysical techniques such as differential scanning fluorimetry, circular dichroism spectroscopy, and size exclusion multi-angle scattering can aid in characterizing the folding pathway, protein stability, and aggregation potential. More advanced techniques such as microscale thermophoresis or NADH-coupled ATPase assays can also be performed in high throughput micro-well formats. By utilizing this high throughput *in vitro* screening pipeline, it will be possible to obtain mechanistically and clinically meaningful information for VUS in HCM.

About 40%–60% of people suffering from HCM have one or more mutations in sarcomere proteins (3, 5, 8). Of the genes with established pathogenicity for HCM (9), the vast majority (35%–60%) are found in the genes encoding for cardiac myosin binding protein C (*MYBPC3*) and myosin heavy chain (*MYH7*) (8, 10, 11) (Figure 1A). Of those, a significant proportion is thought to be due to missense mutations (4%–19% in *MYBPC3%* and 93% in *MYH7*), underlying its substantial contribution to HCM (11–13). Other genes that contribute a smaller proportion of HCM cases include *TNNT2* (5%–10%), *TNNI3* (5%–7%), *MYL2* (2%–4%), *MYL3* (1%–2%), *TPM1* (<1%), and *ACTC1* (<1%) (14). Interestingly, pathogenic substitutions seem to cluster in certain regions of the mutated protein (12, 15–17). For example, HCM-linked missense variants in MYBPC3 have been shown to cluster in specific regions, i.e., domains C3, C6 and C10, suggesting that those domains might be mutational “hot-spots” (12). Similarly, HCM-linked variants in MYH7 cluster in specific regions commonly associated with stabilizing the cardiac myosin head OFF state (i.e., interacting heads motif), in good agreement with the myofilament hypercontractile phenotype of HCM variants (18).

A variety of techniques are used to identify genetic variants, such as whole genome sequencing or targeted HCM multigene panels. The variants are then classified based on criteria developed by the American College of Medical Genetics and Genomics (19, 20). Although whole genome techniques yielded larger numbers of pathogenic variants helping confirm the diagnosis for many (21, 22), it also resulted in an exponential increase in “variants with uncertain significance” (VUS) (23). As a result, there is ambiguity and difficulty in clinical interpretation.

Due to these limitations, VUS are typically disregarded in the clinical decision-making process because there is insufficient information (5, 24). However, prior research showed that sarcomere mutations of uncertain significance or multiple VUS variants in an individual with HCM are associated with earlier disease onset and worse outcomes, thus, improved VUS characterization is critical for clinical management and improved outcomes (23, 25).

Here, we provide a roadmap of validated basic science methods and emerging concepts to help reclassify VUS and address the current limitations of VUS interpretation. These methods can improve characterization of HCM-associated VUS by obtaining molecular, mechanistic, and functional information, thereby, aid in risk stratification, improved medical management and prognostication. Finally, characterization of the pathogenicity or mechanisms of VUS will facilitate development of targeted disease-modifying therapies. These methods can be applied to research in dilated cardiomyopathy (DCM), restrictive cardiomyopathy (RCM), arrhythmogenic cardiomyopathy (ACM), or other genetic cardiomyopathies.

## Computational and genomic methods used in identification of VUS

The classification of mutations in people with HCM starts with pooling data from population, disease and sequence databases and *de novo*, allelic, computational, predictive, or segregation data (19, 26). Gene-level experimental tools for high throughput screening of identified HCM mutations for pathogenicity, are reviewed elsewhere (27–29). Although large databases like the ClinVar database (https://www.ncbi.nlm.nih.gov/clinvar/), SHaRe registry https://www.theshareregistry.org/), and HGMD database (https://www.hgmd.cf.ac.uk/ac/index.php) have been created and curated by the above-mentioned computational methods, large numbers of HCM mutations are still classified as VUS and require re-classification (9, 25, 30).

Many computational tools were developed to improve the prediction of the pathogenicity of genetic variants in recent years (31–33). CardioBoost utilizes an algorithm called “disease-specific variant classifier” to predict the pathogenicity of missense variants of inherited cardiomyopathies and arrhythmias (34). The authors showed a high level of accuracy for variants classified with >90% confidence, which were associated with disease status and clinical severity (34). In fact, disease-specific classifiers have been shown to perform better than methods not trained specifically on features specific to the genes involved in HCM (35). A similar machine learning algorithm using the etiological fraction showed that 4%–20% of cases could be reclassified into pathogenic variants and be used for clinical applications and predictive testing in probands’ relatives (36).

Other tools utilize high-resolution structural data of proteins and the effect that mutations have on protein folding and stability to predict their pathogenicity (37). A study of people with *MYBPC3* VUS using the STRUM tool (evaluating the change in free energy of domain folding upon introduction of a mutation) showed that mutations that produced misfolding were associated with lower event-free survival (38).

Developments in neural networks and artificial intelligence also allow identification of pathogenicity in cardiac sarcomere protein mutations. For example, the disease mutation, phenotype, and pathogenicity in cardiac myosin and myosin binding protein C (MyBPC) mutations were combined to predict global disease mechanism using a neural/Bayes network (39). Although there are limitations in AI technology, work has been done to overcome those challenges and aid in a robust characterization of the functional consequences of VUS and the interpretation of variant classification (40).

Because many computational algorithms have not been validated, the relative performance in identifying potential pathogenicity of variants were assessed in a recent study (11). The authors developed a method to perform variant prediction benchmarks and quantified which algorithms were better in discriminating HCM variant pathogenicity than others (11). They reported that utilizing a combination of the best performing tools can help to narrow down the most important VUS to screen (11).

As there are many different computational tools (11, 32), it may be beneficial to create a consolidated platform of all available algorithms to streamline the in-silico re-classification process, and potentially produce a combined score of pathogenicity scaled by the tested accuracy of each tool. The combine use of these rapid computational algorithms may improve accuracy of prediction and help guide clinical practice.

## Rapid protein modeling and molecular dynamics simulations

With improvements in various aspects of molecular dynamics simulations (MDS) such as modeling software, high performance computing, or advanced sampling techniques, MDS can be readily applied to mutational analyses of VUS in HCM (41–44) (Figure 1A). In the protein simulation workflow, one of the most time-consuming processes is model creation and validation. A recently developed tool (“Ensembler”) may enable a high throughput method to produce simulation-ready ensembles of protein models with and without VUS mutations (45). It can accomplish the series of tasks necessary to build a validated model by combining various tools and libraries including homology modeling, refinement, protonation, solvation, and simulation using open-source Python codes that can also be customized (45).

Following the modeling process, many techniques can be applied to study HCM VUS pathogenicity. One method is simply to simulate two models, one with and without the VUS of interest, and to compare the results of protein structure, dynamics, or interactions. However, this method can be time consuming and require much user input. Thus, many automated or semiautomated servers and tools have been developed to accelerate the process. In the Galaxy server, one can rapidly assess hydrogen bond interactions and principal components (transforming higher dimensional data to a set of orthogonal axes) to determine how intra- and inter-molecular structure and dynamics are affected by the VUS (46). In the tool HTMD, more detailed parameters such as relaxation or equilibrium time scales, folding/unfolding pathways, standard free energy, protein conformation, and secondary structure changes can be screened (47). Other high throughput MDS methods and algorithms assess the mechanism or kinetics of protein-ligand association and modulation by amino acid substitutions (48).

The automation process provides valuable information about a HCM VUS rapidly, but it will still require further study for “hits” or mutations that seem to alter structure or function. For example, a confirmatory MDS study for an HCM-causing substitution in cMyBPC (p.Y235S) showed that this pathogenic variant altered specific intramolecular interactions that explained the hypercontractile cross bridge behavior (41). Another study showed that protein MDS combined with experimental correlation was helpful in reclassification of VUS (49). They used the averaged structural changes resulting from various thin filament protein variants together with differential scanning calorimetry (DSC) experiments to propose the reclassification of nine VUS mutations as benign, likely benign, likely pathogenic, and pathogenic (49). Such combinatorial workflow can provide an additional method to reaffirm disease mechanisms.

Simulations can be time consuming and resource heavy; however, with the continual improvement in the speed of computation, and streamlining processes of simulations, we foresee that in silico modeling and simulation will be a valuable tool in assessing pathogenicity of many HCM VUS.

## High throughput *in vitro* screening tools for rapid characterization of VUS

Although in silico methods for predicting variant pathogenicity have significantly improved over the last decade (50), the output scores or results do not inform about potential molecular etiologies. More biologically meaningful insights into the structural and functional effects of VUS can be gained by utilizing high throughput pipelines for the production, and biophysical and biochemical characterization of a large number of protein constructs (Figure 1B).

Gene synthesis has become an affordable tool for the design and creation of large libraries of protein expression constructs. Subsequent small-scale expression of these constructs in micro-well format not only allows an initial screen for protein folding by measuring the distribution of protein variants in the soluble and insoluble cellular fractions(51), but also generates sufficient material (usually in the low milligram scale) for initial biophysical characterization (52). Hexahistidine-tagged single or multi-domain constructs of sarcomere proteins can be produced in high yields and purified to >90% homogeneity using a single optimized purification step (53), suggesting that the production of large number of protein constructs carrying individual VUS is highly feasible. Recombinant proteins produced from bacterial sources usually do not carry any post-translational modifications (PTMs) identified in the mammalian heart *in vivo* (i.e., serine or threonine phosphorylation). However, known PTMs can be readily introduced into purified protein constructs using *in vitro* biochemical assays (53–55) and incorporated into the analysis pipeline.

Misfolded proteins can derail proteostasis by aggregate-formation, local cleavage or accelerated protein turnover (56). Stability of solubly expressed protein domains can be directly assessed in a high throughput manner via differential scanning fluorimetry (DSF) in either 96- or 384-well format, which allows for the identification of variants that likely alter domain folding by changes in the observable melting temperature (57–60). Initial “hits” in DSF screen can subsequently be confirmed using orthogonal methods such as circular dichroism (CD) spectroscopy, which can give additional information of changes in protein secondary and tertiary structure. More recently, a high-throughput label-free chemical denaturation workflow has been developed that allows the determination of protein thermodynamic stability using a semi-automated plate reader system (52). Lastly, size exclusion-multi angle light scattering (SEC-MALS) in combination with an auto-sampler allows the rapid assessment of the aggregation behavior of large number of protein variants. The combined workflow of solubility screens, and various techniques to assess domain stability and folding will allow the rapid identification of potential pathogenic variants that cause HCM via changes in proteostasis.

Previous studies showed that about a third of investigated VUS in *MYBPC3* do not affect either mRNA or protein stability (61, 62), adding an additional layer of complexity and difficulty to the classification of those variants into either benign or pathogenic. Variants that do not affect mRNA/protein stability are likely to alter protein-protein interactions (39). However, traditional techniques such as isothermal titration calorimetry or surface plasmon resonance spectroscopy severely limit the number of variants that can be examined because they are too time consuming.

This limitation can be overcome by incorporating new biophysical interaction techniques into the *in vitro* screening pipeline. Microscale thermophoresis (MST) is a rapid and sensitive method to quantify biomolecular interactions, which in contrast to classical methods is highly material-, time-, and cost-efficient (63, 64). MST measures the movement of biomolecules along temperature gradients which is determined by the molecule's size and shape, hydration shell and surface charge distribution. Previous studies have successfully used MST to characterize the binding of sarcomere protein domains to both thin and thick filament components (53, 65, 66). Moreover, recent developments in plate reader technologies accelerated the measurement of myofilament protein function by utilizing Foerster Resonance Energy Transfer (FRET)-based technologies to probe protein-protein interactions (67, 68). FRET is based on the radiation-free transfer of energy from a donor to an acceptor fluorophore when they are in sufficient proximity to each other (<15 nm). It can therefore be employed to determine both the structural dynamics and interactions of proteins. Recent studies have successfully employed FRET to test for the effects of HCM-associated mutations on cMyBPC structural dynamics and its interaction with both actin and myosin (68, 69).

Additionally, myofilament function can readily be measured in a high throughput micro-well format using NADH-coupled ATPase assays (70). Previous studies used this assay system to measure the effects of cMyBPC fragments on thin filament activation (71), and protocols can be readily adopted to test for the functional effects of a plethora of VUS in other proteins. Lastly, induced pluripotent stem cells (iPSCs) have diverse applications and are extensively used in genetic studies, but are the topic of other focused reviews and are not reviewed here (72–82).

In summary, high throughput *in vitro* screening pipelines have the potential to not only discriminate between pathogenic and benign variants, but also help to understand the molecular etiologies associated with individual variants (Figure 1). Integration of experimental results into meaningful matrices to assess pathogenicity, and bridging between the structural and functional consequences observed in isolated proteins to cell and organ level function are areas of focus for future improvements.

## Mathematical modeling and simulation to explore effects of VUS on higher level function

Computer modeling may be able to complement the experimental techniques described above and eventually be scaled to test a large number of VUS in short time. The primary goal would be to develop a framework that can predict whether a variant will lead to clinically significant organ-level dysfunction. Once that is accomplished, it might be possible to extend the framework to test potential therapeutic interventions.

Both sarcomere (83–85) and organ-level (85, 86) modeling have rich histories but screening cMyBPC VUS is particularly challenging. In particular, the computer model will need to span from molecular events that occur with timescales of milliseconds to organ-level growth that takes place over weeks and months (87). Numerous challenges will need to be overcome.

We will take cMyBPC as an example. At the sarcomere level, the computer model will need to reproduce the effects of cMyBPC VUS on myofilament level function. At present, cMyBPC is thought to modulate contractile function in two ways: by stabilizing myosin heads in their functional OFF or super-relaxed state, and by extending towards and subsequently binding to thin filaments, which can alter its regulatory state. These competing effects are further complicated by the fact that cMyBPC is localized to distinct regions of thick filaments so that some myosin heads are likely to be directly impacted by cMyBPC while others are left unaffected. Recent structural data suggests that even within the C-zone, each of the three crowns within the thick filament's 43 nm repeat could interact with cMyBPC in a different way (88, 89). Different strategies for simulating these interactions exist (90) but spatially-explicit models that track the location and status of individual molecules in the filament lattice arguably provide the most direct approach (91–93). This area of work remains relatively underdeveloped, but there are published simulations performed using the FiberSim framework that predict how different modes of cMyBPC function will impact myofilament contractile function (94).

Scaling towards the organ level provides additional challenges because of the heart's complex shape and motion during the cardiac cycle. The most common approach is to use finite element modeling, but this technique is very computationally demanding. As a result, most organ-level models are based on very simple contraction modules that are unable to capture the complexity of cMyBPC effects. One approach would be to embed a spatially explicit system like FiberSim (94) inside each element of a complex 3D model, but these calculations will have to be optimized for wide-spread deployment. A simpler alternative is to drive an organ-level model like CircAdapt (95) with a sarcomere-level system that can capture cMyBPC's effects.

One of the remaining challenges is how to simulate growth. By definition, individuals with HCM have abnormally thick ventricular walls. Ideally, the modeling framework would be able to capture that thickening so that benign variants of cMyBPC lead to hearts of normal size while pathogenic mutations produce walls that thicken over time. This is another area of cutting-edge research, and the technology is advancing rapidly (96). Most models to date have used macroscopic variables, such as stress or strain, to drive growth but recent studies (97) suggest that intrinsic sarcomere-level contractility may be a better predictor of wall thickening. Whether eccentric growth (changes in chamber diameter) is paired directly to concentric growth (changes in wall thickness) remains unclear.

**Figure 2:**
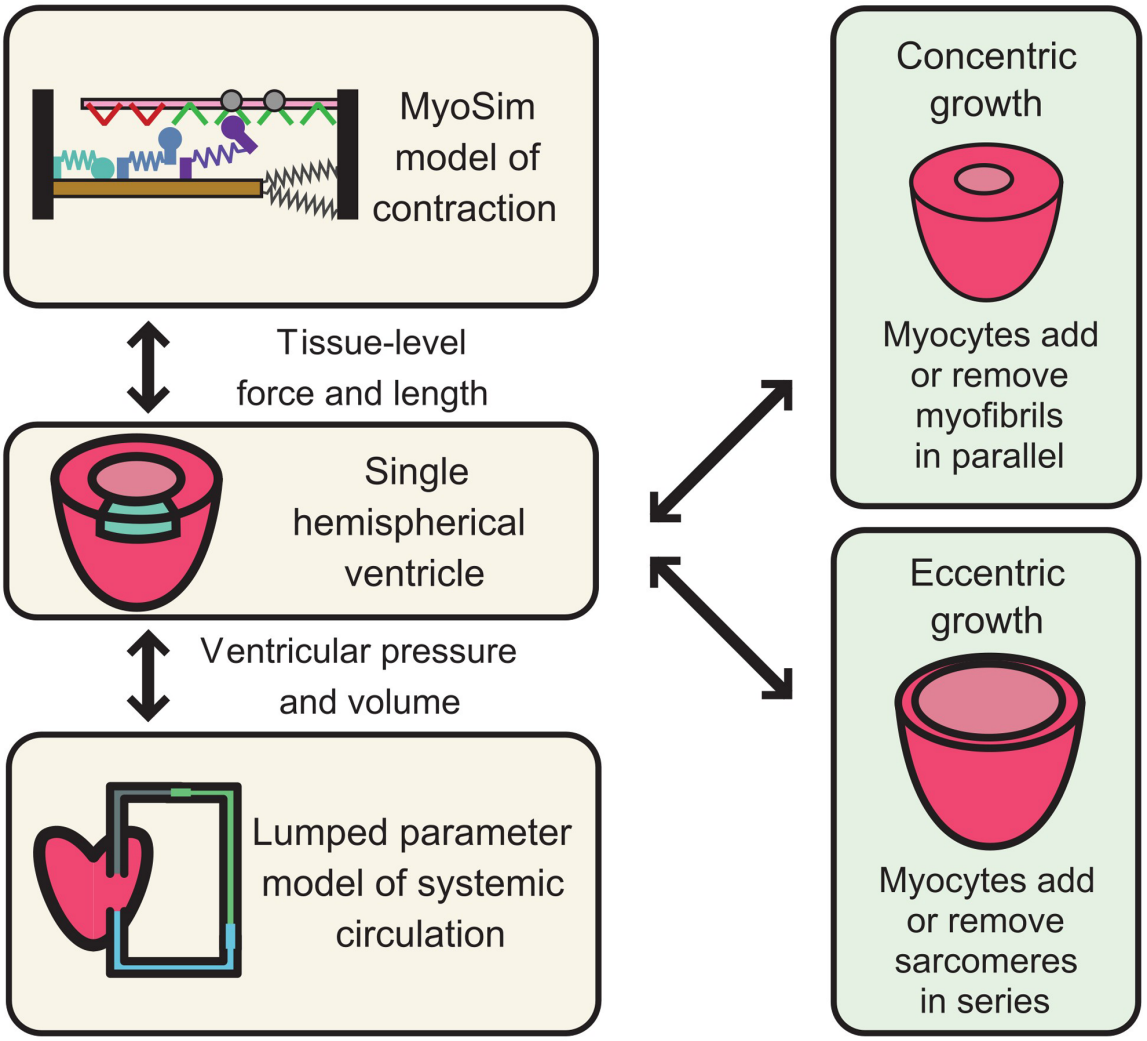
Mathematical modeling and simulation framework for HCM-associated VUS in cardiac MyBPC. The left-hand panels illustrate a multiscale system in which a sarcomere-level model of myofilament function is embedded inside a ventricle and pumps blood around a closed circulation. Growth can be added to this framework by allowing myocytes to add myofibrils in parallel (concentric growth, wall thickening) or to add sarcomeres in series (eccentric growth, chamber dilation). If the approach is to be useful for testing the potential impact of VUS, the sarcomere-level model must reproduce the different potential biophysical actions of cardiac MyBPC.

In summary, computer modeling has the potential to help bridge the gap between genetic variants and predictions of clinically important phenotypes (Figure 2). This will require bridging multiple structural and temporal scales, but important components of the framework already exist at each level. The main challenge will be developing a system that links the disparate scales together.

## Discussion and perspectives

The key issues regarding variants of uncertain significance are the huge numbers of variants identified with genome sequencing technology, difficulty in interpretation, and lack of use in clinical decision making. We reviewed various existing basic science tools and emerging frameworks to address some of these limitations facing VUS interpretation, and how these technologies can be applied to characterize or reclassify HCM-causing VUS. Although there are still many limitations of various algorithms and techniques mentioned, it may provide a conceptual workflow to guide future work in elucidating the functional role of a VUS in HCM. There are other basic science methods not reviewed here that may be applicable as well (e.g., rapid animal model generation and testing) (98–100). By utilizing advanced computational techniques and simulation, high throughput *in vitro* methods, and multi-level mathematical modeling, the improved characterization of HCM VUS will facilitate better medical decision making, improve risk stratification, and allow personalized treatment options. The exploration of VUS causing HCM may also lay the foundation for further detailed *in vivo* functional experiments or clinical trials to evaluate evidence-based therapies.
